# Exploring Political Mistrust in Pandemic Risk Communication: Mixed-Method Study Using Social Media Data Analysis

**DOI:** 10.2196/50199

**Published:** 2023-10-20

**Authors:** Ali Unlu, Sophie Truong, Tuukka Tammi, Anna-Leena Lohiniva

**Affiliations:** 1 Finnish Institute for Health and Welfare Helsinki Finland; 2 Department of Computer Science Aalto University Espoo Finland

**Keywords:** political trust, social media, text classification, topic modeling, COVID-19, Finland, trust, authority, public health outcome, pandemic, perception, mistrust, interaction, Twitter, Facebook, analysis, computational method, natural language processing, misinformation, communication, crisis

## Abstract

**Background:**

This research extends prior studies by the Finnish Institute for Health and Welfare on pandemic-related risk perception, concentrating on the role of trust in health authorities and its impact on public health outcomes.

**Objective:**

The paper aims to investigate variations in trust levels over time and across social media platforms, as well as to further explore 12 subcategories of political mistrust. It seeks to understand the dynamics of political trust, including mistrust accumulation, fluctuations over time, and changes in topic relevance. Additionally, the study aims to compare qualitative research findings with those obtained through computational methods.

**Methods:**

Data were gathered from a large-scale data set consisting of 13,629 Twitter and Facebook posts from 2020 to 2023 related to COVID-19. For analysis, a fine-tuned FinBERT model with an 80% accuracy rate was used for predicting political mistrust. The BERTopic model was also used for superior topic modeling performance.

**Results:**

Our preliminary analysis identifies 43 mistrust-related topics categorized into 9 major themes. The most salient topics include COVID-19 mortality, coping strategies, polymerase chain reaction testing, and vaccine efficacy. Discourse related to mistrust in authority is associated with perceptions of disease severity, willingness to adopt health measures, and information-seeking behavior. Our findings highlight that the distinct user engagement mechanisms and platform features of Facebook and Twitter contributed to varying patterns of mistrust and susceptibility to misinformation during the pandemic.

**Conclusions:**

The study highlights the effectiveness of computational methods like natural language processing in managing large-scale engagement and misinformation. It underscores the critical role of trust in health authorities for effective risk communication and public compliance. The findings also emphasize the necessity for transparent communication from authorities, concluding that a holistic approach to public health communication is integral for managing health crises effectively.

## Introduction

Risk communication is an integrated part of any emergency response that consists of a real-time exchange of information, advice, and opinions between experts, community leaders, officials, and the people who are at risk [1]. Failure to communicate the right message effectively can result in a loss of trust, which in turn can influence compliance with public health measures leading to negative health social, and economic implications [2]. Trust toward authorities is of key importance when people lack the necessary knowledge to evaluate the risk and they need to rely on experts and authorities to interpret the information. Typically, public health officials and other authorities support the public by helping inform their risk judgments, preferences, and choices that in turn can motivate the uptake of public health measures [3-5]. Studies show that during recent infectious disease outbreaks such as Ebola in 2014, H1N1 swine flu in 2009, and the COVID‐19 pandemic, there were correlations between trust in the authorities and the public’s perceived disease severity, perceived virus transmissibility, information-seeking behavior, and willingness to adopt interventions such as recommended hygiene practices and physical distancing measures [6-8]. A lack of trust in the authorities can elevate the spread of misinformation and disinformation including misinterpreted messages, failed warnings, false rumors, and inconsistent information, all of which can negatively influence adherence to public health measures [9].

Trust in the authorities changes based on time and place. Therefore, it is important to monitor it during an emergency. Social listening, a continuous process of collecting web-based and offline data using standard tools, has increased during the pandemic. It can also be used to monitor trust toward authorities. Social listening projects have taken many forms worldwide. Some projects use big data and dashboards to present the findings, such as the World Health Organization Early Artificial Intelligence–Supported Response with Social Listening, which monitors COVID-19–related web-based discussions in 30 countries [10]. Other projects have focused on smaller data sets based on manual internet browser searches and qualitative methods [11,12] or mixed data collection of web-based and offline data [13]. In Finland, the Finnish Institute for Health and Welfare (THL) developed tools that monitored the trust of the public toward authorities during COVID-19 through surveys [14] and qualitative social media analysis [15,16]. The tools increased awareness about public mistrust and mistrust narratives among risk communicators in Finland to help them address the issues accordingly. Factors that are known to increase public trust toward authorities include transparency, and early and frequent communication, which increases public familiarity with the topic and with the communicating body [17-19].

Despite extensive discussions within different disciplines, the concept of trust remains vague, and the trust constructs are diversified [20]. Each discipline focuses on different aspects or features of trust. For example, in medicine trust often implies the doctor-patient relationship, in psychology it implies interpersonal trust, and in sociology, it implies trust in society [21]. Behavioral sciences often treat trust as a trigger for behavior change [22]. This diverse interpretation and use of trust leads to confusion. Furthermore, trust is also often linked with risk perception, which refers to people’s evaluations of hazards to which they are or might be exposed [23]. The dynamics of how trust is linked with risk perception in different settings is not always clear [24].

The THL conducted a qualitative study based on Facebook and Twitter narratives to identify and describe factors linked with pandemic-related risk perception. The purpose of the study was to identify concepts that can be embedded in a digital platform to serve as keywords for risk communicators to conduct social listening during future epidemics and pandemics [25]. The study identified 9 risk-related concepts including trust, which was divided into political trust and societal trust. Concepts linked with political mistrust were of special interest as they can act as alerts to risk communicators to promote trust in the authorities in a timely manner during a crisis. The study identified 12 subcategories linked with political mistrust that included unreliable reporting, erroneous statistics, weak decision-making, slow decision-making, politics-first attitude, economy-first attitude, reducing restrictions too rapidly, loose restrictions, changing recommendations, illogical restrictions, suspicious funding, and pandemic conspiracy [25]. Given that the data originated from the social media platforms of a public health agency with a limited audience and contributors, its scope for generalization is restricted. Additionally, the study used purposeful qualitative sampling and analysis, further limiting the ability to draw generalized conclusions. Therefore, it was deemed essential to validate the concepts of political mistrust through the use of big data.

This study was designed to bridge this existing knowledge gap. Using a big data set spanning 3 years, we analyzed the social media communications between THL and the audience on Facebook and Twitter. The research aimed to answer several key questions: How does political trust fluctuate during a pandemic? In which areas does mistrust accumulate? How does the significance of these topics change over time? Last, to what extent do the outcomes derived from computational methods differ from, or align with, those obtained from qualitative research conducted on the same data set?

## Methods

### Data Collection

The Twitter data were extracted using the *academictwitteR* package [26]. Initially, all THL posts were extracted, and then all tweets mentioning THL were collected. This data set consists of posts, replies, and retweets either made by or directed to THL, or retweeted by THL. The data set spans a period of 3 years, from January 1, 2020 to January 1, 2023. The total number of tweets collected was 66,830. Of these, 34,364 were retweets (51.4%), 28,586 were original posts (42.8%), and 3880 were quoted tweets (5.8%). THL made a total of 9999 tweets during this period, consisting of 8516 original posts (85.2%), 1172 retweets (11.7%), and 311 quoted tweets (3.1%). Specifically, 8712 were in Finnish (87.1%), 727 were in Swedish (7.3%), and 537 were in English (5.4%) For the study design, we only kept followers’ comments and replies to THL in Finnish. Preprocessing involved removing duplicates and retweets, removing tweets with less than 30 characters, and retaining tweets containing COVID or coronavirus keywords, resulting in 3744 unique tweets.

The Facebook data were collected using the Facepager app [27], covering the same time period. However, the Facebook platform has different functionality; once a page owner creates a post, there constant communication starts between followers and page owners. Each can respond to the posts at several levels; however, we only collected the first comment to the THL posts. As a result, we collected 83,074 user comments and 3170 THL posts. As we did in the Twitter data set, we only kept followers’ comments and replies to THL in the Finnish language. In the preprocessing stage, we removed duplicate posts, kept only user comments with a text length greater than 30 characters, removed posts that only included links, and finally filtered the text to retain posts that only included COVID and coronavirus keywords. The final Facebook data set consisted of 9885 unique posts.

In total, 2 data sets were merged, resulting in 13,629 posts. These posts were shuffled, and 4240 of them were randomly selected for the annotation task. Out of these, 3095 were Facebook posts, and 1145 were Twitter posts.

### Ethics Approval

This study adheres to strict ethical guidelines and has received approval (THL/738/6.02.01/2021) from the Ethics Committee at the Finnish Institute for Health and Welfare. All data used are deidentified to ensure anonymity and confidentiality, eliminating the possibility of tracing back to original posts or users. Data storage and use are secured, and findings are used responsibly to contribute to scholarly discourse.

### Text Classification

For text classification, we aim to identify political mistrust from Twitter and Facebook content using computational methods. In the framework of risk perception, trust is defined as a psychological state that encompasses the willingness to accept vulnerability, predicated on positive anticipations regarding the intentions or actions of another entity [3]. Thus, we operationalize mistrust as a negative attitude or attitudes held by an individual toward players in political systems in Finland or elsewhere (ie, individual ministers, parties) and public health institutions (national ones, such as THL and the Finnish Medicines Agency, and international ones, such as the European Centre for Disease Prevention and Control and the World Health Organization, in addition to medical doctors and representatives of media). The code book was developed based on a previous qualitative study, which divided political mistrust into subcategories including unreliable reporting, faulty statistics, weak decision-making, slow decision-making, politics-first attitude, economy-first attitude, reducing restrictions too rapidly, loose restrictions, changing recommendations and restrictions, illogical restrictions, suspicious funding, and pandemic conspiracy [25].

To prepare the training data for the initial stage, we randomly selected 4240 texts from the main data set. In total, 4 research assistants who were native Finnish speakers manually annotated these texts for mistrust categories. Annotators were asked to label a tweet as mistrust, trust, or neutral/unclear stances (representative posts are in Table S1 in Multimedia Appendix 1). For our research, we condensed these label categories into binary classifications after annotation, with a score of 1 for mistrust and a score of 0 for trust or neutral/unclear position. Before annotators were assigned a similar set of 60 text samples to annotate individually, 5 annotation training rounds were conducted. Before each annotation session, we calculated the interrater reliability using Krippendorff α coefficient to measure the degree of agreement among annotators over the given set of data. During annotation training, disagreements about annotations were discussed, and the final labels of these annotations were agreed on through majority voting. After the fifth session, the average Krippendorff α for mistrust was .68; within the established guidelines, a score of 0.8 or higher is considered excellent, a score between 0.67 and 0.8 is deemed good, a score between 0.5 and 0.67 is considered moderate, and a score below 0.5 is considered poor. Therefore, the observed α of .68 indicates good interrater reliability, signifying a satisfactory level of agreement among the coders. Out of 4240 tweets, 2544 (59%) tweets were labeled as normal, and 1756 (41%) tweets reflected mistrust by the manual annotation.

As a state-of-the-art language model, Bidirectional Encoder Representations from Transformers (BERT) is commonly fine-tuned for multiple natural language processing (NLP) tasks, including text classification [28]. Additionally, the BERT model can be trained with different languages. In 2019, an NLP group from Turku University published FinBERT, a BERT-based pretrain language model for the Finnish language [29]. The FinBERT model is reported to have better performance than other popular models, including multilingual BERT, convolutional neural networks, and long short-term memory [30,31].

For the text classification task, we fine-tuned the FinBERT model with the annotated data. By comparing the accuracy of various models that are trained with different parameters and model versions, we identify the best-performing model, which is later used to label the remaining data. On the test data set containing 516 samples, our best-performing model has an accuracy of 80%. Our model has a higher accuracy compared to similar BERT models that are trained to predict public trust in politicians [32].

### Topic Modeling

Once the mistrust contents were identified, we used the topic modeling technique to identify the most representative topics from these contents. BERTopic, a topic-modeling technique [33] using the semantic similarity of BERT embeddings, is used. Recent research suggests that BERTopic stands out by not only generating distinctive topics but also offering novel insights when compared to other models such as Top2Vec, nonnegative matrix factorization, and latent Dirichlet allocation (LDA) [34].

BERTopic algorithm consists of a series of steps to create topic representation. Each step is a separate language model containing a set of tunable parameters [33]. The embedding layer converts text input into numerical vector embedding by using the FinBERT model for the Finnish language. Because vector embeddings tend to have high dimensions, dimensionality reduction, using the UMAP algorithm, is often required to cluster these document embeddings effectively. The hierarchical density-based spatial clustering of applications with noise (HDBSCAN) algorithm is then used to cluster the embedding outputs of the uniform manifold approximation and projection (UMAP) model into sets of similar embeddings to extract topics. Each cluster of embedding is converted into a separate document, from which topic representations can be obtained using the cTF-IDF algorithm.

Due to the modular nature of BERTopic, multiple variations of the topic model can be achieved. Researchers can determine the desired outcome (eg, minimum number of output topics) and create multiple models using different sets of parameters. Output topics of each model are then manually inspected to verify whether they are meaningful and match the desired outcome. In this research, optimal results were achieved with UMAP set at 25 neighbors and 10 components, HDBSCAN clustering with a minimum of 20 documents, and 15 descriptive words per topic.

Furthermore, the best representative documents per topic can be extracted with BERTopic. As a result, researchers can use them to have a better understanding of the topics and subsequently construct a theme connecting similar topics together. In this paper, besides extracting these representative documents corresponding to each topic, we also select other documents from individual topics to manually inspect.

## Results

### Text Classification

Using this fine-tuned model on the rest of the unlabeled data, we identified 5765 samples of mistrust messages (42.3%) and 7864 samples of trust or neutral messages (57.7%). Given that most people who engage with THL on social media do so because they are concerned about COVID-19, a large proportion of the posts are either trustful or neutral toward the THL. However, there is also a significant amount of mistrust, which fluctuates considerably throughout the pandemic.

**Figure 1:**
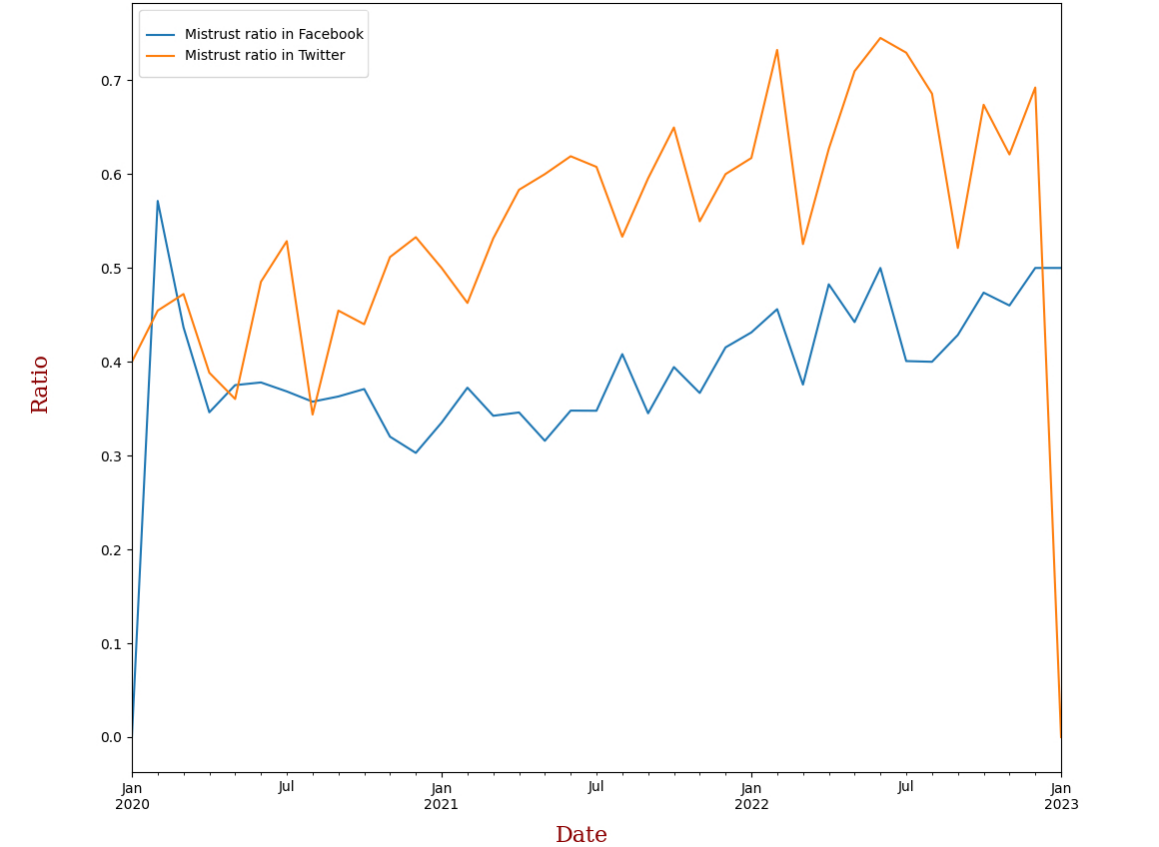
Timeline of mistrust in Facebook and Twitter.

We analyzed the ratio of mistrust messages for each source (eg, Twitter mistrust ratio = total mistrust messages from Twitter / total messages from Twitter) over time (Figure 1). We found that mistrust peaked in early 2020 when the pandemic was spreading rapidly and causing global alarm. However, mistrust declined around April 2020, as people became more accustomed to the situation. We also observed that Twitter users (orange line) expressed more mistrust than Facebook users (blue line) throughout the pandemic. While the Facebook mistrust ratio stayed below 40% until 2022, it rose above it afterward. On the other hand, the Twitter mistrust ratio increased steadily with some fluctuations during the entire period. We attribute this to the presence of malicious bots that targeted THL’s social media accounts with misinformation and antivaccination propaganda (Unlu A, PhD, et al, unpublished data, 2023). As a result, THL suspended its Twitter account at the end of 2022 [35]. Although Facebook has better mechanisms to detect and remove bot accounts [36], our results suggest that THL still faces challenges from these coordinated attacks.

### Topic Modeling

**Figure 2:**
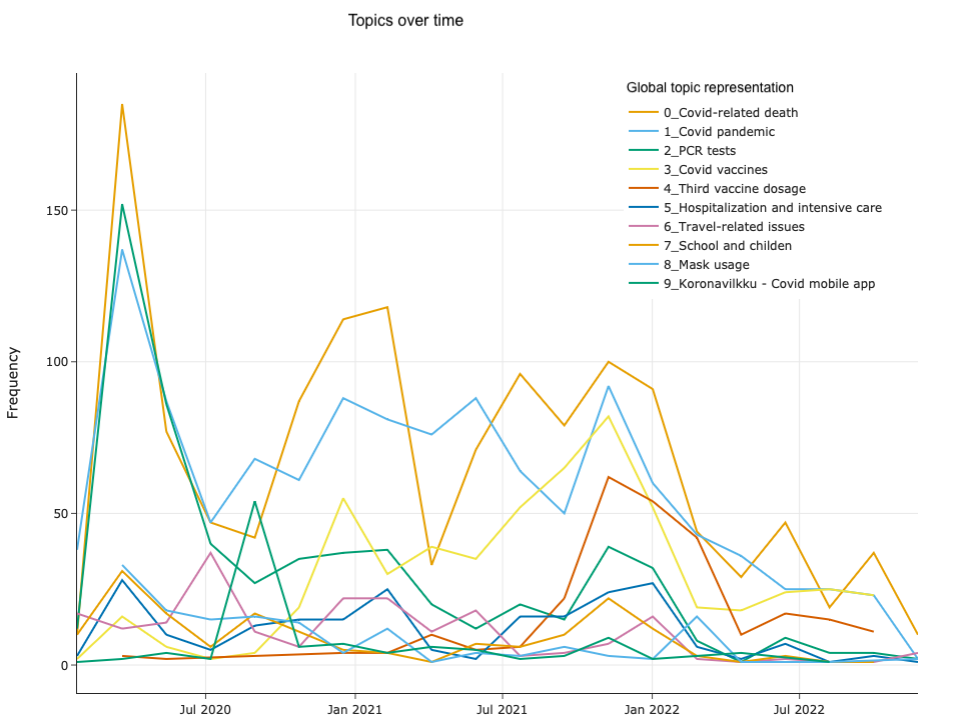
Timeline of top 10 topics during the pandemic. PCR: polymerase chain reaction.

For topic modeling, BERTopic identifies 43 topics in total. The timeline of the 10 most representative topics is shown in Figure 2 (The distribution of tweets across topics is in Multimedia Appendix 1). The main concern for society is the rising number of coronavirus-related deaths, which spiked after certain global events or developments, such as the emergence of new variants of the virus or the need for booster shots. The discussions around death-related issues include understanding the causes of death, questioning the government’s statements on death rates, reporting deaths among vaccinated people, and highlighting the high mortality rate among older people.

The second topic covers the concerns about how to cope with the pandemic at the societal or governmental level. It includes discussions about the strategies to resume normal life, the effectiveness of vaccines in this regard, the comparisons of different vaccine brands, and the inconsistencies in the government’s statements on these matters.

The third topic is about polymerase chain reaction tests, their reliability, and their implications for daily life. The fourth topic is about the effectiveness of vaccines, in terms of reducing death risks, preventing infection, and providing lasting protection. Similarly, the fifth topic focuses on the need for booster doses of vaccines. The third booster shot raises doubts among people about the efficacy of vaccines and some allege that vaccinated people experience serious side effects of these shots. The sixth topic includes discussions about hospitalization and COVID-related illness. Specifically, the discussions revolve around people who brought new variants of the virus to the country, the risks of travelers for hospitalization, and the failure of corona mobile apps to monitor sick people. The seventh topic focuses on travel-related inconveniences, in terms of how travelers contribute to virus transmission.

The eighth topic includes discussions related to school openings and students. Most of the dissatisfaction on this issue stems from the fact that schools remained open throughout the pandemic in Finland. The government was accused of taking insufficient measures at schools, failing to monitor sick students effectively, ignoring the long-term effects of COVID-19 on children, allowing students to be the main source of virus spread, keeping schools open while other countries closed them, and so on.

The ninth topic is about the effectiveness of mask use in preventing virus transmission and the complaints about the lack of clear guidelines on this matter. Finally, the tenth topic is about the mobile app that was used by THL to track the infected people. The majority of the complaints are primarily focused on the technical aspects of the application, such as rapid battery depletion and installation issues across diverse phone brands. Although the relevance of the topics fluctuated over time, our results indicate that their share decreased gradually toward the end of the pandemic.

### Comparative Analysis of Quantitative and Qualitative Research Outcomes

The second component of our study aimed to juxtapose the findings from a previous qualitative study with those derived from our computational method. Given that the prior study was conducted over a relatively shorter time frame, we anticipated that our analysis would unearth a broader array of topics spanning the entirety of the pandemic period, along with their respective trend variations. Simultaneously, we sought to pinpoint minor topics that could be identified via human evaluation but might elude detection by the computational method. To this end, we analyzed the 12 most representative posts for each topic and categorized them as outlined in Table 1.

**Table 1:** Distribution of topics across main themes related to COVID-19.

Main themes	Subtopics
Insufficient information on COVID-19 health effects and complications	Recovery stories and challenges; pandemic persistence and breakthrough infections; gender differences in COVID-19 mortality; influenza that affects PCR^a^ test results; hospitalization and corona-related sickness; PCR test availability and accuracy, and COVID-19 mortality rates and causes
The spread and impact of COVID-19 conspiracy theories and misinformation	Vaccine effectiveness and impact on infection rates; mask effectiveness and misinformation; protectivity and effectiveness of vaccines; vaccine side effects and safety concerns; vaccine misinformation and conspiracy theories; and vaccine booster doses
Social impacts and behaviors	Mask-wearing attitudes and practices; efforts and challenges to fight the epidemic; quarantine experiences, frustrations, and compliance to regulations; and virus transmission modes and prevention methods
Public sector impacts and challenges	Postal service delays and disruptions due to COVID-19; health care access and information; health care disruptions and missed appointments due to COVID-19; government criticism and dissatisfaction; and criticism of Finland’s COVID-19 measures and enforcement
Challenges and controversies in COVID-19 reporting and guidance	English-language tweets about COVID-19; nurse and care-related complaints; reactions to THL’s letter and recommendations; lack of clear and consistent guidelines for COVID-19 prevention; and wastewater and water quality monitoring for COVID-19
Policy choices and challenges	Finland’s pandemic response and evaluation; COVID-19 passport requirements and controversies; Finland’s early easing of restrictions and consequences; and perceptions of vaccine mandates and coercion
The impact of COVID-19 on retail and restaurant sectors	Retail sales trends and web-based shopping during the pandemic; and restaurant industry challenges and COVID-19 safety measures
Travel restrictions and risks	European football championships in St. Petersburg and COVID-19 concerns; Lapland’s COVID-19 situation and tourism; Estonia-Finland travel policies and effects; travel-related risks and virus transmission; and Koronavilkku-related discussions
Country and region-specific issues	Finland’s pandemic response and evaluation; Nordic countries’ COVID-19 comparisons and travel implications; Uusimaa region’s COVID-19 situation and measures; Israel’s COVID-19 situation and vaccination campaign; and Sweden’s COVID-19 situation; strategy; and support for Swedish-speaking Finns

^a^PCR: polymerase chain reaction.

Following an iterative review of the topic contents, we organized them into 9 primary themes. One of these is “insufficient information on COVID-19 health effects and complications.” This theme fundamentally addresses the deficiency or inaccessibility of comprehensive information concerning the health implications and complications associated with COVID-19. It encapsulates the public’s ongoing quest for accurate and detailed information, covering a wide array of subjects. These include the diversity of symptoms exhibited by individuals infected with the virus, the variance in disease severity across different demographic groups, the potential long-term implications for physical and mental health, and the identification of specific complications linked to COVID-19.

Understanding these aspects is instrumental in shaping the public’s perception of the risk associated with the virus. For instance, a lack of comprehensive knowledge about the range of symptoms may hinder early detection and accurate self-assessment of personal risk. The absence of information about how the disease’s severity varies among demographic groups could lead to an underestimation or overestimation of personal risk. The potential for long-term physical and mental health consequences, when not sufficiently communicated, can exacerbate public anxiety and contribute to further mental health challenges. Furthermore, ignorance about specific complications linked to COVID-19 might result in inadequate precautions or a disregard for the disease’s seriousness. Hence, the theme is intertwined with the public’s risk perception. The accessibility and availability of comprehensive, accurate information regarding the health impacts of COVID-19 is paramount in mitigating misconceptions and facilitating a balanced understanding of risk, thereby directly influencing public health outcomes.

The theme titled “spread and impact of COVID-19 conspiracy theories and misinformation” covers a spectrum of subjects. These include the efficacy of vaccines and their influence on infection rates, the effectiveness and misrepresentation of masks, the protective power and efficacy of vaccines, concerns about vaccine side effects and safety, along with the proliferation of vaccine misinformation and conspiracy theories. This category delves into the broad dissemination and repercussions of conspiracy theories and misinformation related to COVID-19.

The theme “social impacts and behaviors” covers an array of topics, which include attitudes and practices toward mask-wearing, discussions about COVID-19 in English-language tweets, efforts, and obstacles encountered in combating the epidemic, experiences related to quarantine along with associated frustrations and compliance with regulations, and knowledge of virus transmission modes and preventive methods. This category examines the social and behavioral facets of the COVID-19 pandemic, with a focus on society’s stance on mask use, the dialogue and sentiment expressed through the English language (mixed-language elements persist despite using a language detection algorithm to clean English posts; often involves English articles clarified in Finnish or vice versa), the diverse initiatives implemented to battle the epidemic, the trials and experiences linked to quarantine measures, and the societal understanding of virus transmission routes and preventative measures. Public trust in political institutions impacts preventive behaviors like mask-wearing, shapes the tone of social media discussions, affects public cooperation in pandemic management efforts, influences quarantine compliance, and determines the acceptance of information about virus transmission and preventive strategies. Thus, political trust significantly shapes societal behaviors and responses during the COVID-19 pandemic.

The theme “public sector impacts and challenges” concentrates on the repercussions and difficulties encountered by the public sector—encompassing governments, health organizations, and public institutions—amid their response to the COVID-19 pandemic. This delves into aspects like postal services, health care, and education, illuminating the intricacies these sectors grapple with during the pandemic. It aims to underscore the adaptive strategies, criticisms, and dilemmas surfacing within these domains, to comprehend and tackle the hurdles confronting professionals and the broader public alike.

“Challenges and controversies in COVID-19 reporting and guidance” addresses the challenges and controversies surrounding the reporting of COVID-19 data and the guidance provided by health organizations and authorities. It examines issues such as data accuracy, the interpretation and presentation of statistics, differing perspectives on public health recommendations, and the public’s trust in official information sources.

“Policy choices” and challenges involve evaluating Finland’s pandemic response, controversies surrounding COVID-19 passports, early easing of restrictions and consequences, perceptions of vaccine mandates, and criticism of Finland’s COVID-19 measures and enforcement. It explores the decision-making process and associated challenges in implementing policies to address the pandemic.

“The impact of COVID-19 on the retail and restaurant sectors” focuses on the specific impacts of the pandemic on the retail and restaurant industries. It examines changes in consumer behavior, such as increased web-based shopping and shifts in spending patterns, the implementation of safety measures in retail and dining establishments, the challenges faced by businesses, and the long-term implications for these sectors.

The theme “travel restrictions and risks” includes discourse on a variety of topics such as concerns about the Euro 2020 football matches also held in Russia, the impact of travel policies between Estonia and Finland, comparisons of the COVID-19 situations among Nordic countries, and the risks related to travel and virus transmission. It scrutinizes the consequences of travel restrictions, the intersection of major sporting events and public health worries, the outcomes of international travel policies, and the deliberations concerning virus transmission amid travel.

“Country and region-specific issues” focus on the unique circumstances and challenges faced by different locations during the COVID-19 pandemic. It includes topics such as Lapland’s COVID-19 situation and its impact on tourism, Israel’s COVID-19 situation and the progress of its vaccination campaign, the COVID-19 situation in the Uusimaa region (capital area) and the measures implemented, as well as Sweden’s COVID-19 situation, strategy, and support for Swedish-speaking Finns. This theme underscores the unique circumstances and challenges encountered by different geographical areas. The influence of political trust on these issues becomes evident when considering the acceptance and efficacy of local responses to the pandemic. Political trust can play a pivotal role in the public’s willingness to accept and adhere to local health measures, such as those implemented in the Uusimaa region. Similarly, trust in political leadership and public health agencies may significantly influence the public’s perception of and participation in vaccination campaigns, as observed in Israel. The intersection between public health measures and specific geographical or cultural contexts, such as Lapland’s tourism industry and the support for Swedish-speaking Finns, can be greatly affected by the level of trust in political institutions. Consequently, political trust can substantially shape regional COVID-19 strategies and responses, indicating a profound connection between political trust and country and region-specific issues during the pandemic.

The reference study [25], using qualitative analysis over a shorter timeframe, discerned 12 themes from the same data set. Our results resonate with theirs on multiple subjects, including unreliable reporting, faulty statistics, weak decision-making, rapid reduction of restrictions, loose restrictions, changing recommendations and restrictions, illogical restrictions, and pandemic conspiracy, albeit with variations in labeling. However, our analysis expands on these topics, encompassing wider subtopics within each category. For example, within the topic of “government criticism and dissatisfaction” (under the main category of “challenges and controversies in COVID-19 reporting and guidance”), our findings include not only unreliable reporting and faulty statistics but also dissatisfaction with transparency, lack of accountability among government authorities, silence on certain issues or events, criticism of a short-sighted vision, and failure to produce and distribute masks to the general public, limiting access to health care workers only.

Contrary to our findings, the topics of “economy-first attitude,” “politics-first attitude,” and “suspicious funding” did not emerge as distinct themes in our analysis. This can be attributed to 2 possible explanations. First, it could be a result of the authors’ interpretation and categorization of topics differing from our approach. Second, it is possible that the frequency of discussions related to these topics was not as prominent compared to other themes, leading them to be embedded within our broader main topics. This suggests that these concepts may be intertwined within the larger themes we have identified rather than being standalone topics.

## Discussion

### Principal Findings

This study, based on a comprehensive analysis of social listening during the COVID-19 pandemic, offers valuable insights into public engagement with pandemic-related topics on Facebook and Twitter. The findings highlight the critical role of trust in effective public health management and underscore the need for strategic communication to foster this trust, thereby informing public health communication and policy.

Our analysis spans the entire duration of the pandemic and identifies 43 topics that emerged as significant during this period, as well as the most prominent ones at different time points. Parallel with the previous studies, our results show that the significant role of trust in public health measures was evident in the relationship observed between trust in authorities and the public’s perceptions of disease severity, virus transmissibility, information-seeking behavior, and willingness to adopt interventions during the COVID-19 pandemic [6-8].

Moreover, our analysis revealed that the patterns of mistrust during the pandemic varied significantly between Facebook and Twitter users. This variation can be attributed to the distinct user profiles and platform features of each social media network, which in turn influenced the susceptibility to misinformation. Specifically, the architecture and user engagement mechanisms on Facebook and Twitter contributed to different patterns of misinformation propagation, thereby underscoring the role of platform-specific characteristics in shaping user behavior and susceptibility to misinformation [37]. Specifically, Twitter is more vulnerable to manipulation by trolls and malicious bots in the Finnish case. The dynamic changes in public attention on various pandemic-related topics revealed through this study indicate that trust can be fostered by tailoring messages to align with public trends and concerns, and by devising platform-specific communication strategies that account for the potential risks and limitations of different platforms.

Given that COVID-19 has resulted in an infodemic where people mainly seek and access information through the internet [38,39], computational methods are essential to handle the large volume of user engagement and provide guidance to public health agencies [40]. Our results demonstrate that the new developments in NLP methods can not only capture the nuances identified by qualitative studies but also several other important topics that require the attention of public health agencies [41,42].

The analysis of topic modeling across a diverse range of themes related to the COVID-19 pandemic provides crucial insights into the complexities of public health communication during this global crisis. The study reveals significant public interest in understanding the health effects and complications of COVID-19, emphasizing the need for clear and accurate information about the disease’s physical impacts, its persistence, and the efficacy of diagnostic tools. Concurrently, the investigation highlights the challenge of managing misinformation, particularly focusing on the spread and impact of COVID-19 conspiracy theories [43,44].

### Misinformation

A lack of trust was found to potentially elevate the spread of misinformation and disinformation, thus negatively influencing adherence to public health measures [9]. This underscores the critical need to ensure public comprehension of key preventive measures and treatments. There is very little evidence to suggest that providing facts alone will stop the spread of misinformation and disinformation. More effective ways to combat misinformation and disinformation include debunking and rebunking [45]. The discontent stemming from the information provided by governmental authorities highlights the importance of effective risk communication. This is crucial not only during the acute phase of a crisis but also in the recovery phase, to preserve public trust and curb the dissemination of misinformation and disinformation.

### Criticism of the COVID-19 Response

The wide-ranging social effects of the pandemic and the complex behavioral responses from the public were another critical area of focus [46]. The strain that the pandemic has placed on public services, leading to significant challenges in service provision [47], was also highlighted. Concurrently, the study revealed a degree of public dissatisfaction with government and public health communications, especially pertaining to the clarity and consistency of messaging about COVID-19.

In the realm of public health policy, the COVID-19 pandemic has brought forth a myriad of complexities, particularly regarding the management of the pandemic and the public’s divergent reactions to distinct policy measures. The intricacies of pandemic management can be attributed to a confluence of factors, including the need for rapid decision-making amidst uncertainty, the requirement for effective communication of these decisions to the public, and the challenge of balancing health priorities with socioeconomic considerations. One of the salient themes that emerged from this situation is the heterogeneous public responses to different policy choices [48]. This heterogeneity reflects the diversity in public attitudes, risk perceptions, and socio-cultural contexts across different populations [49]. For instance, policy choices such as school openings, mask mandates, and vaccination campaigns have been met with varying degrees of acceptance, compliance, and resistance worldwide. These variations in public responses underscore the need for public health policies that are not only scientifically grounded but also socially attuned, taking into account the public’s attitudes, beliefs, and cultural contexts.

### Economic Implications

Another significant theme pertains to the considerable economic disruption triggered by the pandemic. This disruption has been particularly acute in sectors such as retail and restaurants, which rely heavily on in-person interactions and have been severely affected by lockdown measures and social distancing guidelines [50]. The economic impacts of the pandemic are multifaceted, spanning from immediate business closures and job losses to long-term shifts in consumer behavior and market structures [51]. The economic consequences of the pandemic further highlight the need for comprehensive pandemic management strategies that integrate health, economic, and social policies. The global nature of the pandemic and the associated challenges with international travel further illuminated the complexity of the situation.

Importantly, the study illuminated the significance of Finnish contexts in understanding and responding to the pandemic, showcasing a range of experiences and strategies across different geographical regions [52], minority groups [53], or multilocal living conditions [54]. These findings underscore the dynamic nature of the pandemic and highlight the need for context-sensitive approaches in public health communication and pandemic management at local and national levels [55].

The need for comprehensive and accurate information about the physical impacts of COVID-19 and its preventive measures underlines the importance of transparency, a factor known to increase public trust in authorities [6-8]. The spread and impact of COVID-19 conspiracy theories, identified in the analysis of topic modeling, further emphasize the need for early and frequent communication from authorities to increase public familiarity with the topic and the communicating body, thereby mitigating the effects of misinformation and disinformation.

### Limitations

This study has a few limitations. First, the data collection process relied on specific packages and applications, which may not capture all relevant posts. Additionally, the focus on first comments and replies to THL posts may not fully represent the depth of discussions. The use of specific keywords for filtering could lead to the omission of important content. The text classification and topic modeling methods, while advanced, may still be subject to biases and limitations in accurately capturing mistrust and identifying significant topics. Last, merging data from different platforms (Twitter and Facebook) with distinct user behaviors may impact the interpretation of results.

### Conclusions

In conclusion, this study underscores the imperative of building and maintaining public trust in authorities during a health emergency like a pandemic. Effective risk communication, which includes providing accurate information, countering misinformation, and being responsive to public concerns, is key to this endeavor. The findings also highlight the need for context-sensitive approaches in public health communication and pandemic management, considering the diversity in public attitudes, risk perceptions, and socio-cultural contexts across different populations. Further research is needed to identify strategies to foster trust and enhance public engagement with health measures in different sociocultural contexts.

## Appendix

Multimedia Appendix 1 Supplementary information on tweet themes.

## Abbreviations

BERT: Bidirectional Encoder Representations from Transformers
HDBSCAN: hierarchical density-based spatial clustering of applications with noise
NLP: natural language processing
THL: The Finnish Institute for Health and Welfare
UMAP: uniform manifold approximation and projection
