# Supplementary material for: Exploring Political Mistrust in Pandemic Risk Communication: Mixed-Method Study Using Social Media Data Analysis
Source: J Med Internet Res. 2023 Oct 20;25:e50199. doi: 10.2196/50199 (PMC10625074; doi:10.2196/50199)
Supplement: Multimedia Appendix 1 [file jmir_v25i1e50199_app1.docx]

Table S1. Sample tweets of according to their labels (Normal and Mistrust).

| Sample label | Clean text sample | Translated sample |
| --- | --- | --- |
| Normal | USERNAME_TWITTER_1 Onko #thl turvallista? #rippijuhla nyt, jonka järjestäjät sairastivat koronan vuosi sitten. Heillä yksi rokotus. Ovat olleet lähitekemisissä viikon sisällä koronaan sairastuneen kanssa, jonka muut kontaktit karanteenissa. Juhla sisä-ja ulkotiloissa. Vieraina riskiryhmäläisiä. | USERNAME_TWITTER_1 Is #thl safe? #rippijuhla^a^ now, the organizers of which contracted corona a year ago. They have had one vaccination. They have been in close contact with a corona patient within a week, whose other contacts are in quarantine. The party is indoor and outdoor. Guests are people from risk groups. |
| Normal | Antaa rokotevastaisten huudella viimeisiään, kohta ei pääse minnekään ilman koronapassia ja työpaikkakin voi olla vaarassa,jos ei piikkejä oteta. Toivottavasti tulevat pian järkiinsä, vapaus vaatii aina myös vastuuta. | Let the anti-vaxxers scream their last words, soon you won’t be able to get anywhere without a corona passport and your job may be in danger if you don’t take the vaccines. I hope they will come to their senses soon, freedom always requires responsibility. |
| Normal | Minä kävin pitkästä aikaa eilen keskustassa. Apteekissa ei voinut mitenkään välttää koronaa. Siellä istui kaikki vierekkäin tuoleilla. Kun kävin wikkellä niin siellä huomasi hyvin että ihmiset tosiaan yrittää pitää sitä väliä joka paikassa. Ruokapuolella oli ainakin aina ostoskärry edessä ja vielä vähän lisää. Kassa ei liukuhihnalla päästänyt ostoksia eteenpäin niin kauan kun edellinen oli saanut ruokansa pois. | I went to the center yesterday after a long time. There was no way to avoid corona in the pharmacy. There, everyone sat next to each other on chairs. When I went to the Wikke^b^ department store, I noticed very well that people really try to keep distance everywhere. On the food side, at least there was always a shopping cart in front and a little more. The cashier did not let the purchases go on the conveyor belt as long as the previous one had finished his food. |
| Normal | USERNAME_TWITTER_1 Hei. Kuinka monta sairaala ja teho hoidossa olevista on joutunut sinne koronan takia? | Hi. How many hospital and intensive care patients have had to go there because of corona? |
| Normal | Marika kuoliko varmasti koronaan? Vai oliko jo muutenki huonossa hapessa? | Did Marika die of corona for sure? Or was she already in bad condition anyway? |
| Mistrust | Karin Kinkk kotitestit ei tunnistanut perheen koronaa ja taatusti oikein otettu. Myös tuttavilla samaa. | Karin Kinkk’s home tests did not recognize the family’s corona and were guaranteed to be taken correctly. The same with friends. |
| Mistrust | USERNAME_TWITTER_1 Tiedän että näin menetelmä toimii. Mutta tämä kertaa THL on vissiin sotkenut asiaan, ja alistuneet ei ole saanut ilmoitus ennen kuin 5 päivää myöhemmin! Ja mikä tämä on että työntekijät ei saa kertoa että talossa on covid-19? Ei kukaan voi määrätä tällaista. | USERNAME_TWITTER_1 I know this is how the method works. But this time THL must have messed up, and the surrendered people didn’t get the notification until 5 days later! And what is this that the employees are not allowed to tell that there is covid-19 in the house? No one can order this. |
| Mistrust | Alettu puhumaan pitkäkestoisesta koronasta ja monella, Esim, nyt uutisissa TV 1-SSÄ klo.18.00. Jopa joka toisella 😱. Jos noi monella niin pakko olla silloin rokotetuilla eli onko kaikki rokotettuja jolla todettu pitkäkestoiset oireet. Johtuisko rokotteesta itsestään nämä pitkäkestoiset korona oireet. Ettei näillä medioissa ulostuloilla tilasto infot yritä peitellä rokotteen haittavaikutteita. Tuleeko altistuneelle joissaintapauksissa pitkäkestoisia oireita jos on rokote alla. | Started talking about long-Covid and many people, for example, now in the news on TV1 at 18:00. Even every other one 😱. If those many people must have been vaccinated then, that is, are all vaccinated people who have been diagnosed with long-lasting symptoms. Could these long-Covid symptoms be caused by the vaccine itself. That the statistical information coming out in these media does not try to cover up the harmful effects of the vaccine. Will the exposed in some cases have lasting symptoms if he/she already has taken the vaccine. |
| Mistrust | Alkaa olla koronaluvut niin korkeat ettei järjestelmä kykene näyttämään niitä!  Tehdään näennäisiä päätöksiä millä ei ole mitään vaikutusta yhtään mihinkään mutta ollaan tekevinään koronalle jotain:  “Uudenmaan alueellinen koordinaatioryhmä esittää Etelä-Suomen aluehallintovirastolle, että sisätiloissa järjestettävien yhteislaulu- ja yleisötilaisuuksien osallistujamäärää rajoitetaan. Yleisötilaisuuksien osalta rajoitukset koskisivat sellaisia katsomojen osia, joissa on seisomapaikkoja.” | The corona numbers are starting to be so high that the system cannot display them! One makes apparent decisions that have no effect on anything at all, but pretends to be doing something for the corona:  “Uusimaa’s regional coordination group proposes to the Regional Administrative Agency of Southern Finland that the number of participants in joint singing and audience events organized indoors be limited. As for public events, the restrictions would apply to those parts of the stands where there are standing places.” |
| Mistrust | USERNAME_TWITTER_1 Huolestuttavat luvut! Nyt jokaisen pitäisi ottaa #koronaryhti sydämenasiaksemme! Emme kai halua mustaa joulua? | USERNAME_TWITTER_1 Worrying numbers! Now everyone should take #koronaryhti to heart! We don’t want a black Christmas do we? |

^a^rippijuhla: the christian confirmation party at the age of 15.

^b^Wikke is the spoken name of Sokos Wiklund department store in the centre of Turku city, Finland.

Table 2. Proportion of total post volume (n=13488) attributed to each theme.

| Topic | CustomName | Ratio (%) |
| --- | --- | --- |
| 0 | 0_Covid-related death | 9.91 |
| 1 | 1_Covid pandemic | 8.83 |
| 2 | 2_PCR tests | 4.40 |
| 3 | 3_Covid vaccines | 4.21 |
| 4 | 4_Third vaccine dosage | 1.98 |
| 5 | 5_Hospitalization and intensive care | 1.66 |
| 6 | 6_Travel-related issues | 1.56 |
| 7 | 7_School and childen | 1.25 |
| 8 | 8_Mask usage | 1.12 |
| 9 | 9_Koronavilkku - Covid mobile application | 0.83 |
